# Complete Genome Sequence of *Corynebacterium imitans* DSM 44264, Isolated from a Five-Month-Old Boy with Suspected Pharyngeal Diphtheria

**DOI:** 10.1128/genomeA.01210-14

**Published:** 2014-11-20

**Authors:** Sabrina Möllmann, Andreas Albersmeier, Christian Rückert, Andreas Tauch

**Affiliations:** Institut für Genomforschung und Systembiologie, Centrum für Biotechnologie (CeBiTec), Universität Bielefeld, Bielefeld, Germany

## Abstract

The complete genome sequence of the type strain *Corynebacterium imitans* DSM 44264 comprises 2,565,321 bp with a mean G+C content of 64.26%. The detection of the antibiotic resistance genes *erm*(X), *aphA1-IAB*, *strA*-*strB*, and *cmx* is fully consistent with the previously observed multidrug-resistant pattern of *C. imitans* isolates.

## GENOME ANNOUNCEMENT

*Corynebacterium imitans* was first isolated in February 1996 from a 5-month-old boy suffering from acute respiratory disease that was initially diagnosed as pharyngeal diphtheria (1). Several adults who had contact with either the young boy or among each other developed similar symptoms of a respiratory disease that was treated successfully with antidiphtheria antitoxin (1). A polyphasic taxonomic study of four multidrug-resistant *C. imitans* strains isolated from four different patients revealed that this bacterium represents a new subline within the genus *Corynebacterium*, loosely associated with *Corynebacterium afermentans* and *Corynebacterium mucifaciens* (1, 2). Since then, *C. imitans* has been recovered very rarely from human clinical material, including five blood culture isolates (3), two isolates from urine specimens of women undergoing overactive bladder treatment (4), and two strains from unknown clinical samples (5, 6). To gain insights into the gene repertoire of this pathogenic corynebacterium and the molecular basis of its antibiotic resistances, we sequenced the genome of the type strain *C. imitans* DSM 44264.

*C. imitans* DSM 44264 was obtained from the Leibniz Institute DSMZ (Braunschweig) and was grown in brain heart infusion broth-yeast extract medium at 37°C (7). Genomic DNA was purified with the MasterPure Gram-positive DNA purification kit after treatment of the bacterial cells with Ready-Lyse lysozyme (Epicentre). A sequencing-ready DNA library of *C. imitans* was generated with the Nextera DNA sample preparation kit (Illumina). The DNA library was sequenced in a 2 × 300 nucleotide paired-end run using the MiSeq reagent kit version 3 (600 cycles) and the MiSeq desktop sequencer (Illumina), resulting in 2,206,453 paired reads and 526,240,527 detected bases. The paired reads were assembled with the Roche GS De Novo Assembler software (release 2.8) to yield 25 contigs in 11 scaffolds. The scaffolds were ordered with the r2cat tool (8). The remaining gaps in the genome sequence were closed by PCR assays with the BIOTAQ DNA polymerase (Bioline). The finishing step of the genome project was facilitated by the Consed tool (version 26) (9).

The chromosome of *C. imitans* DSM 44264 has a size of 2,565,321 bp with a mean G+C content of 64.26%. The annotation of the genome sequence was performed with the NCBI Prokaryotic Genome Annotation Pipeline and the GeneMarkS+ software (version 2.6), revealing 2,013 protein-coding regions, 12 pseudogenes, 54 tRNA genes, 1 noncoding RNA gene, and 4 rRNA operons. The previously detected antimicrobial resistance pattern of *C. imitans* (1) is consistent with the presence of typical corynebacterial resistance genes (10, 11), including the azithromycin, erythromycin, and clindamycin resistance gene *erm*(X), the kanamycin resistance gene *aphA1-IAB*, the streptomycin resistance genes *strA*-*strB*, and the chloramphenicol exporter gene *cmx*. The aminoglycoside and chloramphenicol resistance genes are located on a complex mobile element that was also identified in *Corynebacterium striatum* (10), *Corynebacterium resistens* (11), and *Corynebacterium urealyticum* (12, 13). The annotation of secreted proteins indicated that the cell surface of *C. imitans* DSM 44,264 is composed of adhesive pili of the SpaDEF type (14) and an exceptional repertoire of cell envelope proteins that might be relevant for pathogen-host interactions.

### Nucleotide sequence accession number.

This genome project has been deposited in the GenBank database under the accession no. CP009211.
